# Liver Haemorrhagic Syndrome in Broilers: Its Effect on Serum Parameters, Antioxidant Capacity, Liver Enzymes and Fatty Acid Profile of Liver

**DOI:** 10.1002/vms3.70372

**Published:** 2025-05-06

**Authors:** Ismail Yavaş, Shahram Golzar Adabi, Necmettin Ceylan

**Affiliations:** ^1^ Department of Animal Science, Faculty of Agriculture Ankara University Ankara Türkiye

**Keywords:** antioxidant capacity, broiler, fatty acid profile, liver haemorrhage, liver enzymes, serum parameters

## Abstract

This study sought to evaluate the occurrence of various degrees of liver haemorrhagic syndrome (LHS) in broilers subjected to commercial rearing conditions. The objective was to investigate the influence of lesion scores on various biochemical parameters. The prevalence of liver lesions graded from 0 to 5 was 12%, 31%, 33%, 15%, 6% and 3%, respectively. The crude protein content decreased, whereas the crude fat content increased in Scores 4 and 5 (*p* < 0.05). The observed increase in thiobarbituric acid reactive substances (TBARSs), carbonyl and sulphydryl levels in Score 5 (82.8%, 43.3% and 74.8%, respectively) suggests that this score may be more susceptible to oxidation compared to livers with no LHS incidence (*p* < 0.01). A reduction in total protein in Scores 4 and 5, a decline in albumin in Score 5, an elevation in uric acid and gamma‐glutamyl transferase (GGT) in Scores 4 and 5 as well as a rise in cholesterol and alanine transaminase (ALT) in Score 5 were noted (*p* < 0.05). Furthermore, there was an increase in aspartate transferase (AST) levels in 3, 4 and 5 (*p* < 0.01). A comparison of the antioxidant data indicated a significant enhancement in the levels of superoxide dismutase (SOD), glutathione peroxidase (GSH PX) and total antioxidants in Scores 1–3, compared to the control group. The lowest levels of SOD, GSH PX and total antioxidants were observed in livers scored 4 and 5 (*p* < 0.01). Besides, an increase in total saturated fatty acids and a decrease in total monounsaturated fatty acid (MUFA), *n*‐6, *n*‐3 and polyunsaturated fatty acid (PUFA) levels were observed in livers scored 4 and 5 when compared to the other groups (*p* < 0.05). In conclusion, this study has demonstrated that elevated levels of LHS scores are associated with a detrimental impact on the physicochemical and oxidative quality of livers and blood serum.

## Graphical abstract

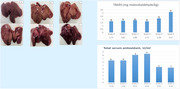

## 1 Introduction

Among producers of animal protein sources, chicken production is one of the most important industries for human nutrition. Broiler production is expected to grow up to 13% worldwide by 2030 (OECD‐FAO 2021). Combined with high demands, genetic selection for rapid growth and the subsequent definition of precise nutritional requirements for chicks are inevitable. These have led the industry to produce modern commercial chicken lines with better feed conversion ratios and body weights, accompanied by changes in body composition, especially higher body fat (Baéza and Le Bihan‐Duval 2013; Golzar Adabi and Demirok Soncu 2019), which may affect metabolic disorders in poultry species. As an appendage of the digestive system, the liver has essential functions and responsibilities in the body, including the metabolism of nutrients, such as fat, carbohydrates, protein, vitamins and minerals, excretion and detoxification processes. The liver regulates the production, storage and release of carbohydrates, lipids and proteins in the metabolism and digestion of nutrients (Denbow 2000). Therefore, research emphasizes a comprehensive study of the metabolic functions of the liver and any dysfunctions that may lead to liver impairment in order to maintain a normally functioning organ and the production of healthy chicken meat (Zaefarian et al. 2019).

Fatty liver haemorrhagic syndrome (FLHS) is well known as a metabolic problem in commercial laying hen flocks, sometimes being fatal in high‐producing laying flocks. It can also spread to broiler flocks as liver haemorrhagic syndrome (LHS). On the other hand, various factors, such as anti‐nutritional factors (e.g., NSP, phytate), toxic substances (e.g., mycotoxins), chemicals (e.g., heavy metals), drugs (e.g., antibiotics), stress factors (e.g., temperature changes) and disease (e.g., LHS caused by hepatitis E virus), can negatively affect the function and health of the liver (Jensen et al. 1974; Akers and Denbow 2013; Shini 2014; Su et al. 2018; Zaefarian et al. 2019). LHS, characterized by excessive fat loading of the liver and abdominal cavity, leads to liver rupture, haemorrhage and, in the acute form, sudden death (Crespo and Shivaprasad 2003). In some birds, oxidized fat accumulates in the liver, leading to lethal haemorrhages (Leeson 2007). Clinical signs of LHS include lethargy and apathy, paralysis and lying on the chest with the neck extended (Butler 1976). It has also been reported that LHS is characterized by swollen and pale livers, liver cells distended with fat vacuoles and bleeding of various sizes (Zaefarian et al. 2019). In addition, fatty liver can give way to metabolic syndrome and diseases, which in turn affect the growth and productive performance of the birds (Moradi et al. 2013; Shini et al. 2019). Studies have found that, especially in broiler nutrition, higher feed intake and restricted activity may reduce broiler production as a result of excessive energy intake or imbalanced energy–protein ratio (Attia et al. 2018; Dey et al. 2018). Liver rupture with subsequent intra‐abdominal haemorrhage is considered the second most prevalent traumatic lesion, with liver rupture and fracture together accounting for approximately 4% of all deaths from all causes (Kittelsen et al. 2015).

There are some strategies on the agenda to maintain liver health status and protect it from LHS in broilers (Sonkusale et al. 2011; Marimuthu et al. 2019; Malisorn et al. 2020). The above highlights the fact that maintaining liver health in broilers is equally important from two perspectives: First, the health and welfare of broilers and, second, the production and consumption of healthy chicken by‐products. Given the significance of LHS in broilers and its effects on post‐slaughter liver and serum parameters, further research seems essential and indispensable. As a result, the current study aimed primarily to first identify the occurrence of haemorrhagic lesions in broilers and then to evaluate the effects of different scoring samples on serum chemistry, antioxidant status and serum liver enzymes, lipid–protein oxidation and fatty acid profile in the liver.

## 2 Materials and Methods

### 2.1 Determination of LHS Prevalence

**FIGURE 1:**
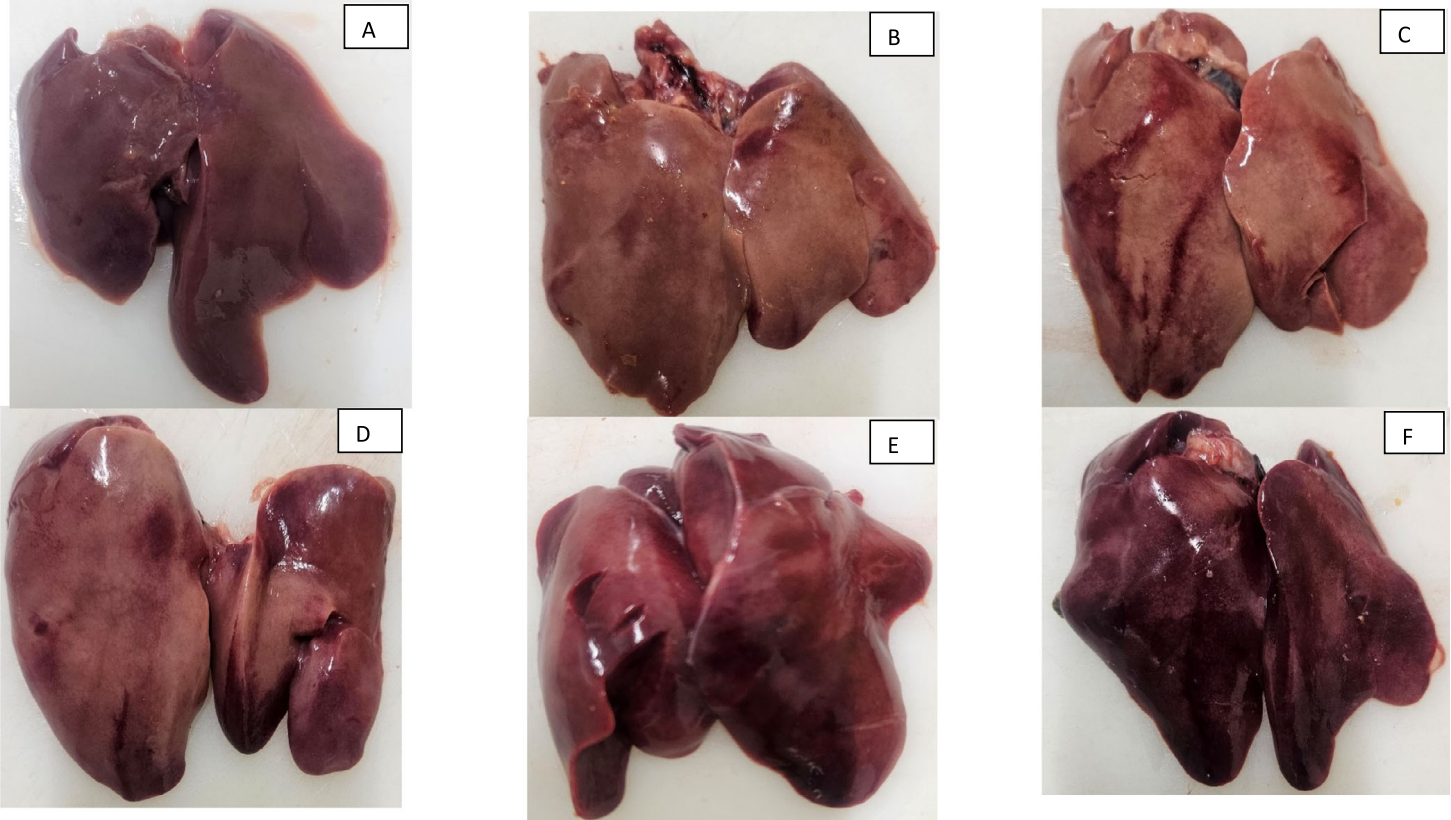
Livers of broiler chickens with different scores of LHS (petechial haemorrhages, haematomas and massive haemorrhages) (A)–(F) present scores from 0 (normal liver without any lesion) to 5 (severe haemorrhage).

To assess the occurrence of liver lesions by lesion scoring, samples were collected from broiler integrations from June 2019 to July 2022. After examining the livers, the lesions, if present, were scored from 0 to 5 (Shini et al. 2019). The severity of haemorrhage was graded on a scale ranging from 0 to 5 (see Figure 1) on both the dorsal and ventral surfaces of the liver. A score of 0 indicated the absence of haemorrhages. Score 1 represented up to 10 subcapsular petechial or ecchymotic haemorrhages. Score 2 denoted more than 10 subcapsular petechial or ecchymotic haemorrhages. Scores 3–5 corresponded to large haematomas and massive liver haemorrhage accompanied by rupture of the liver capsule. A haemorrhagic score ranging from 3 to 5 was considered to be highly indicative of LHS (Shini et al. 2019). The incidence of each score was 12%, 31%, 33%, 15%, 6% and 3%, respectively, based on scores from 0 to 5.

### 2.2 Sampling Procedure for Laboratory Experiment

Each liver score was analysed for proximate composition, lipid and protein oxidation, fatty acid profile, blood biochemistry and antioxidant status. Mycotoxin levels in all ingredients used in broiler diets were analysed (data not shown). For the present study, a total of 200 male ROSS 308 broilers fed the same diet were selected from 12,000 birds at 41 days of age. For serum collection, blood was collected by venipuncture from the basilic vein of chicks into serum vacutainers with gel as a clotting activator. Blood samples were taken from all 200 birds and labelled accordingly. Following the determination of the liver scores, each sample was subsequently matched with its corresponding score. Sera were separated by centrifugation at 4500 rpm for 15 min and transferred to Eppendorf tubes for further analysis. Then birds were slaughtered by cutting the jugular vein. At the same time, after evisceration in a commercial slaughterhouse, liver samples were lesion‐scored for haemorrhages. Twenty livers from each score were then selected, frozen and stored at −20°C until further analysis.

### 2.3 Proximate Analysis of Liver

Moisture (950.46), crude fat (Soxhlet procedure, 991.36), crude protein (Kjeldhal method, 955.04) and crude ash (920.153) of homogenized samples were determined according to AOAC (2010).

### 2.4 Lipid and Protein Oxidation Analysis

Lipid peroxidation as thiobarbituric acid reactive substances (TBARSs) and protein oxidation for carbonyl and sulphydryl were performed as described by Mielnik et al. (2006), Soyer et al. (2010) and Srinivasan and Hultin (1997), respectively. TBARS values were expressed as mg malondialdehyde (MDA)/kg liver. Carbonyl and sulphydryl levels were calculated as nmol/mg protein.

### 2.5 Serum Analysis

Total protein, albumin, uric acid, total cholesterol, aspartate transferase (AST), gamma‐glutamyl transferase (GGT), alanine transaminase (ALT), superoxide dismutase (SOD), glutathione peroxidase (GSH PX) and total antioxidant levels in serum were analysed using commercial kits (Pars Azmoon Co. Tehran, Iran and Novin Navand Salamat Pishtaz Co., Urmia, Iran).

### 2.6 Fatty Acid Profile

A cold extraction method was used to extract the fat content from livers. As previously reported by the International Union of Pure and Applied Chemistry (1987), after the preparation of fatty acid methyl esters (FAMEs) from crude fat, the methyl esters were injected into a GC (Shimadzu GC‐2010, Shimadzu Co., Kyoto, Japan) equipped with a flame ionisation detector and a DP23 capillary column (30 m × 0.25 mm; 0.25 µm, Agilent J&W, CA, USA). The samples of 1 µL were manually injected at a 1:100 split, and the fatty acid profiles were subsequently determined through a comparison of the retention times with FAME Mix C8‐C24 (Supelco 18,918‐1AMP; Sigma Interlab). Each acid was expressed as a relative percentage of the total fatty acids. The GC operating conditions were as follows: The temperature was initially set at 40°C for 5 min, then at a rate of 3°C per minute until it was eventually stabilized at 190°C, which was maintained for 20 min. The injector and detector temperatures were set at 230°C and 240°C, respectively. Helium was the carrier gas, with a flow rate of 0.5 mL/min.

### 2.7 Statistical Analysis

All analyses were performed by one‐way ANOVA procedure SAS software (SAS 2001). Tukey's post hoc test was used to compare treatments’ means, and the level of significance was set at *p* < 0.05. All data are shown as mean values ± SD.

## 3 Results

### 3.1 LHS Incidence in Broiler Integration

The incidence of LHS score in broilers at 41 days of age was determined on the basis of data collected from integrations. The incidence of LHS in broilers scored 0–5 was found to be 12%, 31%, 33%, 15%, 6% and 3%, respectively, at 41 days of age. The highest incidences belonged to Scores 1 and 2, with 31% and 33%, respectively, whereas Scores 0, 4 and 5 were associated with the lowest incidences, with 12%, 6% and 3%, respectively. In addition, high‐grade lesions (Score 5) were found in 3% of liver samples, whereas no lesions (Score 0) were found in 12% of liver samples. The risk factor scores for the chickens can be categorized as follows: The incidence of LHS was found to be no risk in approximately 12% with Score 0, low risk in approximately 64% with Scores 1 and 2, serious risk in approximately 21% with Scores 3 and 4 as well as severe risk in approximately 3% with Score 5 in all liver samples examined.

### 3.2 Proximate Composition of Liver Samples

The results of liver weight to body weight and proximate analysis of liver samples are presented in Table 1. The dry matter and crude ash content of the livers did not show significant (*p* > 0.05) differences among the different LHS scores. In comparison to Scores 0–3, which exhibited the lowest fat and the highest protein contents, Score 5 demonstrated the highest fat and the lowest protein levels (Table 1; *p* < 0.05). These findings demonstrated a correlation between the severity of LHS and the composition of the liver, whereby a rise in the level of crude fat was accompanied by a decrease in the level of crude protein, particularly as the score increased from 3 to 5. Consequently, there was a significant increase in the level of fat by 12.42%, whereas the amount of protein decreased significantly by 1.74% in birds with a Score of 5 compared to birds with no liver lesion (Table 1; *p* < 0.05).

**TABLE 1:** Comparison of proximate composition of broiler livers with different liver haemorrhage lesion scores (*n* = 20/liver haemorrhagic syndrome [LHS] score).

Parameters	Score 0	Score 1	Score 2	Score 3	Score 4	Score 5	*p* value
Liver weight, % of BW	2.27 ± 0.02	2.30 ± 0.07	2.26 ± 0.08	2.26 ± 0.12	2.28 ± 0.09	2.33 ± 0.08	ns
Dry matter, %	24.19 ± 1.19	24.35 ± 0.44	24.36 ± 0.57	24.15 ± 1.02	24.34 ± 1.18	24.07 ± 1.16	ns
Crude fat, %	11.92 ± 0.86^b^	12.05 ± 0.74^b^	12.11 ± 0.74^b^	12.08 ± 0.62^b^	12.95 ± 0.47^ab^	13.40 ± 1.03^a^	^*^
Crude protein, %	68.76 ± 0.47^a^	68.82 ± 0.32^a^	68.89 ± 0.57^a^	68.74 ± 0.46^a^	67.60 ± 0.71^b^	67.56 ± 0.35^b^	^*^
Crude ash, %	5.64 ± 0.50	5.72 ± 0.38	5.83 ± 0.77	5.73 ± 0.50	5.47 ± 0.49	5.81 ± 0.55	ns

*Note*: All data are shown as mean values ± standard error of the mean (SEM).

Abbreviation: ns, not significant.

^a,b^Means within the same row without common superscripts are significantly different.

^*^ *p* < 0.05.

### 3.3 Lipid and Protein Oxidation

The effect of liver haemorrhage score on TBARS (mg MDA/kg) level, which is an indicator of lipid oxidation, and carbonyl and sulphydryl, which are indicators of protein oxidation, are summarized in Table 2. The statistical analysis revealed that birds with a score of 0–4 exhibited similar TBARS, carbonyl (nmol/mg protein) and sulphydryl (nmol/mg protein) values (*p* > 0.05). Despite an increasing trend in protein oxidation in terms of carbonyl and sulphydryl from Scores 0 to 4, no significant changes could be observed (Table 2; *p* > 0.05). The TBARS (*p* < 0.01), carbonyl (*p* < 0.05) and sulphydryl (*p* < 0.01) concentrations in livers with a score of 5 were found to be significantly higher, at 83.2%, 43.3% and 77.7%, respectively, in comparison to normal livers (Table 2).

**TABLE 2:** TBARS (mg malondialdehyde/kg), carbonyl (nmol/mg protein) and sulphydryl (nmol/mg protein) values of broiler livers with different liver haemorrhage lesion scores (*n* = 20/liver haemorrhagic syndrome [LHS] score).

Parameters	Score 0	Score 1	Score 2	Score 3	Score 4	Score 5	*p* value
TBARS	1.73 ± 0.33^b^	1.81 ± 0.56^b^	1.89 ± 0.50^b^	1.72 ± 0.17^b^	2.18 ± 0.44^b^	3.17 ± 1.04^a^	^**^
Carbonyl	50.16 ± 18.75^b^	50.89 ± 3.40^b^	52.00 ± 4.78^ab^	53.07 ± 3.32^ab^	62.97 ± 5.50^ab^	71.89 ± 3.86^a^	^*^
Sulphydryl	7.41 ± 2.24^b^	7.60 ± 2.69^b^	8.82 ± 2.28^b^	10.13 ± 4.69^ab^	11.02 ± 4.86^ab^	13.17 ± 5.86^a^	^**^

*Note*: All data are shown as mean values ± standard error of the mean (SEM).

^a,b^ Means within the same row without common superscripts are significantly different.

** *p* < 0.01,

* *p* < 0.05.

### 3.4 Serum Analysis

The results of serum biochemistry, liver enzymes and antioxidant capacity are shown in Table 3. The results showed that birds with Score 5 had the lowest levels of total protein and albumin (*p* < 0.01; Table 3). In contrast to birds with Score 0 (*p* < 0.05), the level of serum uric acid was significantly higher in birds with Scores 4 and 5 at 53.59% and 68.71%, respectively (Table 3), but there was no significant difference between the other treatments in this respect.

**TABLE 3:** Serum biochemistry, liver enzymes and antioxidant capacity values of broiler with different liver haemorrhage lesion scores (*n* = 20/liver haemorrhagic syndrome [LHS] score).

Parameters	Score 0	Score 1	Score 2	Score 3	Score 4	Score 5	*p* value
Total protein, g/dL	3.40 ± 0.63^a^	3.50 ± 0.54^a^	3.46 ± 0.77^a^	3.02 ± 0.61^ab^	2.77 ± 0.50^b^	2.25 ± 0.38^c^	^*^
Albumin, g/dL	1.54 ± 0.21^a^	1.52 ± 0.21^a^	1.42 ± 0.23^ab^	1.31 ± 0.13^bc^	1.30 ± 0.19^bc^	1.20 ± 0.12^c^	^*^
Uric acid, mg/dL	3.90 ± 0.59^b^	3.77 ± 0.52^b^	3.74 ± 0.47^b^	4.30 ± 0.46^b^	5.99 ± 0.98^a^	6.58 ± 0.66^a^	^*^
Total cholesterol, mg/dL	127.56 ± 6.64^c^	127.68 ± 6.77^c^	127.86 ± 4.13^c^	131.53 ± 4.84^bc^	133.68 ± 3.93^ab^	136.40 ± 3.24^a^	^*^
AST, U/L	274.06 ± 41.30^b^	275.20 ± 56.3^b^	278.50 ± 50.9^b^	337.68 ± 24.05^a^	351.85 ± 27.29^a^	358.85 ± 29.79^a^	^*^
GGT, U/L	13.40 ± 3.29^b^	13.55 ± 4.66^b^	13.90 ± 4.35^b^	17.06 ± 4.52^ab^	18.56 ± 4.94^a^	20.17 ± 3.99^a^	^*^
ALT, U/L	17.44 ± 3.06^c^	17.48 ± 3.33^c^	18.86 ± 3.60^c^	19.57 ± 3.16^bc^	22.12 ± 3.76^ab^	24.12 ± 3.43^a^	^*^
SOD, U/mL	92.59 ± 4.69^c^	92.60 ± 4.70^c^	102.74 ± 7.33^b^	113.86 ± 9.46^a^	77.70 ± 7.07^d^	76.35 ± 4.77^d^	^*^
GSH PX, µmol/L	669.40 ± 33.51^b^	670.03 ± 37.02^b^	746.06 ± 34.08^a^	765.52 ± 27.82^a^	616.12 ± 40.28^c^	602.51 ± 33.30^c^	^*^
Total antioxidant, U/mL	6.49 ± 1.39^b^	6.49 ± 1.66^b^	8.12 ± 1.39^a^	8.44 ± 1.48^a^	4.56 ± 1.26^c^	4.36 ± 0.99^c^	^*^

*Note*: All data are shown as mean values ± standard error of the mean (SEM).

^a,c^ Means within the same row without common superscripts are significantly different.

* *p* < 0.05.

The highest serum AST (U/L) levels were found in Scores 3–5 with 337.68, 351.85 and 358.85 U/L, respectively (Table 3; *p* < 0.05). In addition, the level of GGT (U/L) increased in accordance with the severity of LHS; accordingly, GGT increased significantly (*p* < 0.05) by 38.51% and 50.53% in birds with scores of 4 and 5 (18.56 and 20.17 U/L) compared to livers with a score of 0 at a level of 13.40 U/L. Scores 0–3 had the lowest ALT (17.44, 17.48, 18.86 and 19.57 U/L), and the highest ALT (U/L) was observed in birds at high risk of LHS (24.12 U/L; Table 3; *p* < 0.05).

Although antioxidant parameters, including serum SOD (U/L), GSH PX (µmol/L) and total antioxidants (U/mL), increased significantly in livers scored 1–3, the same parameters decreased in livers scored 4 and 5 (Table 3; *p* < 0.05). For example, the amount of SOD significantly increased by 10.96% and 22.97% in Scores 2 and 3 (102.74 and 113.86 U/mL) and decreased by 16.09% and 17.53% in Scores 4 and 5 (77.70 and 76.35 U/mL) compared to livers with Score 0 at a level of 92.59 U/mL (Table 3; *p* < 0.05).

### 3.5 Fatty Acid Profile

The effect of different haemorrhagic scores on the fatty acid profile of the liver is shown in Table 4. Compared to the other scores, low oleic acid (omega‐9), the major monounsaturated fatty acid (MUFA) in animal tissues, was also found to be most significant (*p* < 0.01) in Score 5 (18.66% in Score 5 vs. 21.46% in Score 0). Linoleic acid and arachidonic acid, two important omega‐6 FAs, were significantly lower by 13.77% and 26.01% in the livers of birds with Score 5 compared to birds with normal livers (Score 0) (Table 4; *p* < 0.01). Total omega‐6 levels were also significantly lower in the livers of birds with Scores 4 and 5 (Table 4; *p* < 0.05). With increasing severity of lesions in the liver, the amount of α‐linolenic acid declined significantly from 0.41% in Score 0 to 0.30% in Score 5 (Table 4; *p* < 0.05). The lowest levels of linoleic acid, linolenic acid, eicosapentaenoic acid (EPA), docosapentaenoic acid (DPA) and docosahexaenoic acid (DHA) were found in livers with Scores 4 and 5 (*p* < 0.05). For example, the levels of EPA, DPA and DHA, known as the omega‐3 fatty acids, decreased significantly by 43.85%, 34.54% and 32.31%, respectively, at Score 5 compared to Score 0 (Table 4; *p* < 0.01). The results showed that livers with Score 5 significantly had the highest amounts of palmitic acid (*p* < 0.01), stearic acid (*p* < 0.05), total saturated fatty acid (SFA) (*p* < 0.01) and n6:n3 ratio (*p* < 0.01) and the lowest amounts of total MUFA (*p *< 0.05), n6 (*p* < 0.01), n3 (*p* < 0.01) and polyunsaturated fatty acid (PUFA) (*p* < 0.01). Like Score 5, n6, n3 and PUFA were also low in Score 4 (*p* < 0.01).

**TABLE 4:** Fatty acid profile (%) of broiler livers with different liver haemorrhage lesion scores (*n* = 20/liver haemorrhagic syndrome [LHS] score).

Fatty acid	Molecular formula	Score 0	Score 1	Score 2	Score 3	Score 4	Score 5	*p* value
Myristic acid	C14:0	0.35 ± 0.02	0.35 ± 0.02	0.35 ± 0.03	0.36 ± 0.04	0.35 ± 0.02	0.38 ± 0.03	ns
Palmitic acid	C16:0	21.78 ± 0.95^c^	21.74 ± 0.97^c^	21.91 ± 1.01^c^	22.16 ± 1.94^c^	25.62 ± 2.15^b^	28.58 ± 1.72^a^	^**^
Stearic acid	C18:0	20.74 ± 1.26^b^	20.60 ± 1.01^b^	20.89 ± 1.72^b^	20.20 ± 1.33^b^	22.54 ± 1.27^a^	22.49 ± 2.07^a^	^*^
Palmitoleic acid	C16:1(*n*‐7)	1.64 ± 0.11	1.65 ± 0.19	1.56 ± 0.17	1.69 ± 0.12	1.57 ± 0.28	1.72 ± 0.14	ns
Oleic acid	C18:1(*n*‐9)	21.46 ± 0.93^a^	21.23 ± 0.65^a^	21.41 ± 1.35^a^	21.09 ± 0.96^a^	20.52 ± 1.51^a^	18.66 ± 0.79^b^	^**^
Vaccenic acid	C18:1(*n*‐7)	0.07 ± 0.02	0.07 ± 0.01	0.07 ± 0.02	0.08 ± 0.02	0.07 ± 0.01	0.08 ± 0.01	ns
Eicosenoic acid	C20:1(*n*‐9)	0.51 ± 0.08	0.49 ± 0.06	0.52 ± 0.10	0.49 ± 0.07	0.46 ± 0.09	0.48 ± 0.09	ns
Linoleic acid	C18:2(*n*‐6)	18.51 ± 1.21^a^	18.29 ± 1.01^ab^	18.19 ± 0.95^ab^	18.25 ± 1.43^ab^	16.51 ± 1.01^bc^	15.96 ± 1.63^c^	^**^
Arachidonic acid	C20:4(*n*‐6)	11.80 ± 0.82^a^	11.52 ± 0.67^a^	11.04 ± 0.71^a^	11.48 ± 0.93^a^	9.03 ± 0.91^b^	8.73 ± 1.78^b^	^**^
Adrenic acid	C22:4 (*n*‐6)	0.61 ± 0.02^a^	0.60 ± 0.05^a^	0.58 ± 0.09^a^	0.61 ± 0.16^a^	0.45 ± 0.13^ab^	0.38 ± 0.08^b^	^**^
α‐Linolenic acid	18:3(*n*‐3)	0.41 ± 0.05^a^	0.39 ± 0.03^ab^	0.39 ± 0.04^ab^	0.40 ± 0.08^ab^	0.33 ± 0.05^bc^	0.30 ± 0.04^c^	^**^
Eicosapentaenoic acid	C20:5(*n*‐3)	0.57 ± 0.06^a^	0.55 ± 0.07^a^	0.52 ± 0.04^a^	0.55 ± 0.05^a^	0.35 ± 0.04^b^	0.32 ± 0.05^b^	^**^
Docosapentaenoic acid	C22:5(*n*‐3)	0.55 ± 0.08^a^	0.54 ± 0.05^a^	0.53 ± 0.05^a^	0.54 ± 0.08^a^	0.40 ± 0.06^b^	0.36 ± 0.06^b^	^**^
Docosahexaenoic acid	C22:6(*n*‐3)	1.64 ± 0.10^a^	1.63 ± 0.11^a^	1.62 ± 0.31^a^	1.57 ± 0.24^a^	1.28 ± 0.15^b^	1.11 ± 0.10^b^	^**^
SFA	N/A	42.87 ± 1.34^c^	42.68 ± 1.34^c^	43.16 ± 1.58^c^	42.71 ± 2.25^c^	48.51 ± 1.93^b^	51.45 ± 1.82^a^	^**^
MUFA	N/A	23.67 ± 1.33^a^	23.45 ± 1.71^a^	23.56 ± 1.53^a^	23.35 ± 1.16^a^	22.62 ± 1.45^b^	20.94 ± 1.26^b^	^*^
*n*‐6	N/A	30.92 ± 1.34^a^	30.41 ± 1.30^a^	29.80 ± 0.92^a^	30.35 ± 1.93^a^	25.99 ± 1.31^b^	25.07 ± 1.94^b^	^**^
*n*‐3	N/A	3.16 ± 0.18^a^	3.11 ± 0.17^a^	3.05 ± 0.27^a^	3.06 ± 0.26^a^	2.36 ± 0.14^b^	2.09 ± 0.10^b^	^**^
PUFA	N/A	34.08 ± 1.39^a^	33.52 ± 1.34^a^	32.85 ± 1.10^a^	33.40 ± 2.02^a^	28.35 ± 1.36^b^	27.16 ± 1.96^b^	^**^
*n*‐6:*n*‐3	N/A	9.80 ± 0.98^c^	9.79 ± 0.62^c^	9.83 ± 0.73^c^	9.98 ± 0.92^bc^	11.06 ± 0.73^ab^	12.02 ± 1.03^a^	^**^

*Note*: All data are shown as mean values ± standard error of the mean (SEM).

Abbreviations: MUFA, monounsaturated fatty acids; N/A, not applicable; PUFA, polyunsaturated fatty acids; SFA, saturated fatty acids.

Abbreviation: ns, not significant.

^a,c^ Means within the same row without common superscripts are significantly different.

^*^ *p* < 0.05.

^**^ *p* < 0.01.

## 4 Discussion

The results of the current study showed that significantly low protein and high fat content could be detected as the degree of LHS increased in the liver; however, the same result could not be found for the content of moisture and ash, as their content did not change. According to several reports, hens with FLHS suffer from lipid metabolic disorders, inflammation and oxidative stress leading to liver dysfunction (Shini 2014; Lv et al. 2018; Xing et al. 2020; Yao et al. 2022). In addition, it is suggested that exposure to FLHS leads to excessive fat accumulation in the liver and abdominal cavity (Crespo and Shivaprasad 2003). In line with our study, Shini (2014) mentioned that the occurrence of FLHS is the result of the abnormal accumulation of fat in the liver, for example, the level of fat can increase up to three times in turkeys suffering from hepatic lipidosis (Visscher et al. 2017). In addition, researchers have positively correlated the high percentage of fat in the liver or abdominal area with high‐energy and low‐protein‐adjusted diets in broilers. Butler (1976) suggested that excessive fat in the liver mainly resulted from an increase in lipogenesis, rather than being absorbed from dietary lipids. Other research has emphasized the role of high‐energy diets balanced with cereals used to meet energy requirements and feed processing (exp. fermentation) on liver fat concentration (Jensen et al. 1976; Pearson et al. 1978; Maurice and Jensen 1978). Specifically, it is argued that high‐energy and low‐protein diets may increase fatty acid ingestion and promote lipid synthesis, giving way to the occurrence of metabolic stress through the accumulation of triacylglycerol and diglyceride, defined as lipotoxic molecules (Choi et al. 2012; Ullah et al. 2019).

Oxidative stress is another contributing cause of lipid accumulation and liver injury through complex signal transduction pathways leading to chemical, sensory and textural deterioration in the liver (Gu et al. 2021). Although the rate of lipid oxidation in tissues is usually determined using the TBARS assay, for protein oxidation it is done by assessing the enhancement of the carboxylation process and the loss of thiol groups (Lund et al. 2011; Guyon et al. 2016). In our study, livers with Score 5 were found to be significantly susceptible to lipid and protein oxidation. As stated by Wu et al. (2021), a higher level of lipid peroxidation in the liver is the sign of oxidation caused by FLHS.

Plasma metabolite and liver marker parameters are useful tools for assessing metabolic changes in organs and tissues. Many researchers have agreed that the measurement of plasma enzymes constitutes a pragmatic approach to the evaluation of FLHS (Rozenboim et al. 2016; You et al. 2023). Furthermore, studies have indicated that enzyme activities, particularly those of AST, ALT and GGT, with GGT proving to be the most sensitive in mammals and birds (Shini 2014), are indicative of liver damage in birds and underscore the importance of measurement. The underlying cause of FLHS is the disruption of lipid metabolism, resulting in symptoms such as haemorrhage and hepatic steatosis. Elevated levels of liver enzymes, such as AST and ALT, can serve as indicators of liver injury and thus represent a critical aspect of the diagnosis of FLHS (Rozenboim et al. 2016; Wang et al. 2023). In the present study, the highest serum AST levels were found in Scores 3–5. The results showed an increase (*p* < 0.05) in uric acid and GGT levels in Scores 4 and 5 and in total cholesterol and ALT levels in Score 5. You et al. (2023) recorded significantly higher (*p* < 0.01) serum levels of total cholesterol, ALT and AST in birds suffering from FLHS compared to their control group. Higher serum ALT, AST, triglycerides, cholesterol, glucose and LDH concentrations have also been documented by other researchers in affected birds (Sabir et al. 2014). In a broiler study, chicks exposed to FLHS also had high cholesterol, triglyceride, HDL and LDL concentrations, whereas in contrast to the current study, where serum protein and albumin concentrations decreased with increasing liver haemorrhage score, no changes were reported for the so‐called parameters/factors (Sonkusale et al. 2011).

Our results showed that in addition to a significant (*p* < 0.05) increasing trend in serum SOD, GSH PX and total antioxidant levels from Scores 1 to 3, there was a significant decreasing trend for the same parameters at Scores 4 and 5. These results suggest that LHS, a stressor factor, has a regressive effect on the antioxidant capacity in the body, further exposing the tissues to reactive oxygen and free radicals (Gu et al. 2021). On the other hand, high levels of MDA (an end product of lipid peroxidation), carbonyl and sulphydryl (end products of protein oxidation) in the liver of birds suffering from LHS indicate further adverse effects of LHS on the overall antioxidant capacity of the body. These results are consistent with those found by Gu et al. (2021) in their study of liver metabolism and antioxidant capacity in laying hens of different ages.

The liver of poultry has been described as a major organ of fatty acid synthesis (Scanes and Braun 2013). In the current study, higher SFA and *n*‐6:*n*‐3 ratios and lower MUFA and PUFA percentages were measured with increasing haemorrhagic levels of liver samples. The relevance of arachidonic acid, EPA and DHA in liver metabolism and inflammation cannot be overlooked (Visscher et al. 2017; Meng et al. 2021). The concentration of these fatty acids decreases with the onset of hepatic steatosis, just as it did in our study: a significant decrease with increasing haemorrhagic score. In addition, on the basis of our results, the level of palmitic acid, which is introduced as a toxic fatty acid for the liver (Yamada et al. 2015), increased in birds with hepatic lipidosis. In line with our study, Visscher et al. (2017) also reported lower palmitic acid in healthy liver samples. Studies have reported impaired and low fatty acid beta‐oxidation in birds with FLHS (Miao et al. 2021), and more recently, You et al. (2023) presented results showing shorter lipid chain length and unsaturation in these birds. In addition, they showed a significant decrease in the content of acylcarnitine with 1 and 2 unsaturation in FLHS laying hens compared to normal birds (*p* < 0.05).

## 5 Conclusion

LHS has emerged as a significant concern within the poultry industry. The present study observed elevated liver haemorrhagic scores at Levels 4 and 5, along with the presence of high concentrations of liver crude fat and low concentrations of liver crude protein. Furthermore, the study revealed that lipid and protein oxidation parameters, TBARS, carbonyl and sulphydryl, exhibited the highest levels of detection at the Level 5 liver haemorrhagic score.These observations were accompanied by increased levels of AST, GGT and ALT and decreased levels of SOD, GSH Px and total antioxidant parameters. The results of this study indicate that examined serum biochemistry, liver enzymes and antioxidant capacity parameters in different lipid species of the liver are manifested in relation to LHS. This may provide insights into the pathogenesis of LHS from different perspectives.

## Author Contributions

Shahram Golzar Adabi designed the trial. Necmettin Ceylan submitted the ethics clearance application. Shahram Golzar Adabi, Necmettin Ceylan and Ismail Yavas executed the trial. Shahram Golzar Adabi and Ismail Yavas collected the data. Shahram Golzar Adabi ran the lab analysis. Shahram Golzar Adabi and Necmettin Ceylan did the statistical analyses. Shahram Golzar Adabi and Necmettin Ceylan interpreted the results. Shahram Golzar Adabi and Ismail Yavas wrote the draft paper. All authors checked the results and contributed to preparing the manuscript.

## Ethics Statement

The authors confirm that the ethical policies of the journal, as noted on the journal's author guidelines page, have been adhered to and the appropriate ethical review committee approval has been received. “The US National Research Council's guidelines for the Care and Use of Laboratory Animals” were followed.’

## Conflicts of Interest

The authors declare no conflicts of interest.

## Data Availability

The authors have nothing to report.
